# Effects of warming on the structure of aquatic communities in tropical bromeliad microecosystems

**DOI:** 10.1002/ece3.9824

**Published:** 2023-02-22

**Authors:** Melissa Progênio, Pablo A. P. Antiqueira, Felipe R. Oliveira, Bianca R. Meira, Fernando M. Lansac‐Tôha, Luzia C. Rodrigues, Gustavo Q. Romero, Liam N. Nash, Pavel Kratina, Luiz F. M. Velho

**Affiliations:** ^1^ Programa de Pós‐graduação em Ecologia de Ambientes Aquáticos Continentais Universidade Estadual de Maringá (UEM) Maringá Paraná Brazil; ^2^ Programa de Pós‐Graduação em Ecologia, Instituto de Biologia (IB) Universidade Estadual de Campinas (UNICAMP) Campinas São Paulo Brazil; ^3^ Departamento de Biodiversidade, Evolução e Ambiente Universidade Federal de Ouro Preto (UFOP) Ouro Preto Minas Gerais Brazil; ^4^ Núcleo de Pesquisas em Limnologia, Ictiologia e Aquicultura Universidade Estadual de Maringá (UEM) Maringá Paraná Brazil; ^5^ Departamento de Biologia Animal, Instituto de Biologia (IB) Universidade Estadual de Campinas (UNICAMP) Campinas São Paulo Brazil; ^6^ School of Biological and Behavioural Sciences Queen Mary University of London London UK

**Keywords:** alpha and beta diversity, climate warming, detritus resources, freshwater ecosystems, natural microecosystems, neotropical

## Abstract

Freshwaters are among the most vulnerable ecosystems to climate warming, with projected temperature increases over the coming decades leading to significant losses of aquatic biodiversity. Experimental studies that directly warm entire natural ecosystems in the tropics are needed, for understanding the disturbances on aquatic communities. Therefore, we conducted an experiment to test the impacts of predicted future warming on density, alpha diversity, and beta diversity of freshwater aquatic communities, inhabiting natural microecosystems—Neotropical tank bromeliads. Aquatic communities within the tanks bromeliads were experimentally exposed to warming, with temperatures ranging from 23.58 to 31.72°C. Linear regression analysis was used to test the impacts of warming. Next, distance‐based redundancy analysis was performed to assess how warming might alter total beta diversity and its components. This experiment was conducted across a gradient of habitat size (bromeliad water volume) and availability of detrital basal resources. A combination of the highest detritus biomass and higher experimental temperatures resulted in the greatest density of flagellates. However, the density of flagellates declined in bromeliads with higher water volume and lower detritus biomass. Moreover, the combination of the highest water volume and high temperature reduced density of copepods. Finally, warming changed microfauna species composition, mostly through species substitution (*β*_repl_ component of total beta‐diversity). These findings indicate that warming strongly structures freshwater communities by reducing or increasing densities of different aquatic communities groups. It also enhances beta‐diversity, and many of these effects are modulated by habitat size or detrital resources.

## Graphical abstract

Effects of warming on structure of aquatic communities.

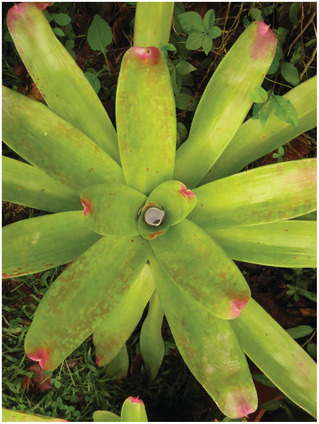

## 1 INTRODUCTION

Climate warming is increasingly impacting natural ecosystems across the globe (Batt et al., 2017; Sala et al., 2000). Climate change may alter the freshwater landscapes in the global context, affecting the storage and redistribution of water bodies and consequently increasing frequency and magnitude of droughts, floods, and sea level rise, reflecting directly on food security, water availability, and human wellbeing (Tapley et al., 2019). Climate change is considered to be one of the greatest threats to human health, affecting pathogen–vector–host systems, particularly over temperate, peri‐arctic and arctic areas, and high‐altitude regions in the tropics (Caminade et al., 2019; Ryan et al., 2019). These changes have an effect on local climate adaptations and interspecific interactions, and also on the current and future distributions of species, especially those living in more vulnerable areas, as well as relying on intraspecific differences in climate tolerance (Razgour et al., 2019).

Multiple freshwater ecosystems have already suffered from the increase in global temperature, resulting in species distribution, feeding, and reproduction rates, among others (IPCC, 2022). This can lead to high estimates of biodiversity loss in freshwater environments, especially in tropical biomes where the percentage of threatened and extinct species is highest (Isbell et al., 2022). This is partly driven by the high sensitivity of freshwaters organisms to climate change, caused by their limited dispersal capacity, high dependence on external physicochemical conditions and water availability, and exposure to multiple, compounding anthropogenic stressors (Ormerod et al., 2010; Woodward et al., 2010). Ongoing climate warming is predicted to trigger complex but poorly understood interactive effects on aquatic biodiversity, particularly on microbial food webs (Zingel et al., 2018). The effects of warming on aquatic food webs can affect species composition and productivity, in addition to direct effects on biochemical and physiological rates that are linked to energetic processes, which influence fundamental processes for ecosystem functions and services (Gårdmark & Huss, 2020; Ohlberger et al., 2011).

The rising threat of climate warming makes full understanding of the impacts of temperature on multiple components of freshwater diversity critically important. This includes responses of both alpha (i.e., local number of species) and beta (i.e., variations in species composition among communities or ecosystems) diversity (Podani & Schmera, 2011; Whittaker, 1972). High beta diversity reflects large differences in composition between local communities within a habitat, and this depends on multiple different processes (Busse et al., 2018). An effective way to study the ecological mechanisms underlying biodiversity responses to stressors is to partition the total beta diversity into its two components: replacement (species substitution) and richness differences (Podani & Schmera, 2011). Substitution is when the number of species remains the same, but the identity of the species changes, while richness difference is characterized by varying species numbers across different communities. Both, substitution and richness difference are governed by mechanisms related to environmental filters, such as climate warming, but to a different degree. Thus, depending on the influence of the stressor on aquatic biota, the two beta diversity components would play complementary roles in structuring ecological communities. Changes in alpha diversity are closely related to the beta diversity (Whittaker, 1960) and a loss of alpha diversity from climate warming could enhance beta diversity, due to the increased dissimilarity among sites with different thermal conditions (Antiqueira, Petchey, & Romero, 2018).

Aquatic communities play a key role in freshwater ecosystems but are often overlooked in studies investigating the impacts of climate warming on alpha and beta diversity. This group is composed of autotrophic microflora (e.g., green and blue algae, diatoms, and myxotrophic flagellates) and heterotrophic microfauna (e.g., testate amoebae, ciliates, copepods, cladocerans, and rotifers). Temperature strongly influences cell chemical composition, nutrient uptake, CO_2_, and growth rates for each algae species (Singh & Singh, 2015). Warming can alter the dynamics of phytoplankton at the ecosystem, community, and population levels. At the ecosystem level, warming can alter the energy balance because in the short term, respiration rates increase more sharply with temperature than photosynthesis, thus warming can act as both a stressor and a driver of physiology (Yvon‐Durocher et al., 2017). At the community level, phytoplankton can be affected by an indirect effect of temperature as a function of their functional characteristics (Machado et al., 2019). And at the population level, warming may favor some phytoplankton groups over others (Lewington‐Pearce et al., 2019), such as cyanobacteria that have higher optimal growth temperatures (Kosten et al., 2012). In relation to microfauna, such as copepods, because they are ectotherms with short generation times, the increase in temperature can quickly affect diversity directly through the influence on the metabolic rates of individuals and indirectly on the abundance and diversity of the population (Rombouts et al., 2009). Microfauna are important indicators of environmental quality because of their rapid responses to environmental change, widespread distribution across all freshwater ecosystems, high population densities (Radhakrishnan & Jayaprakas, 2015), reproductive rates, and trophic niche diversity (Madoni & Bassanini, 1999). Moreover, the microbial loop is critical for ecosystem functioning and tropical aquatic microorganisms are responsible for a larger fraction of the carbon flux than temperate microorganisms (Sarmento, 2012). Yet very little is currently known about microbial community structure, particularly in tropical aquatic environments (Elmoor‐Loureiro et al., 2022; Lau et al., 2019). Previous studies from tropical biomes have largely focused on aquatic macroinvertebrates or vertebrates, leaving a knowledge gap on hyperdiverse groups of aquatic communities (Torres‐Alvarado et al., 2019).

Empirical evidence of the impacts of warming on aquatic communities is often limited to observational studies with low replication or simplified, artificially assembled communities in laboratory experiments. However, freshwater communities occupying small water bodies trapped between bromeliad leaf‐axils, or ‘phytotelmata’ are becoming widely used to test the impacts of environmental change on entire communities and ecosystems (Antiqueira, Petchey, Piccin, et al., 2018; Antiqueira, Petchey, & Romero, 2018; Kratina et al., 2017; Srivastava et al., 2004). Their small size means they can be easily manipulated and controlled such as laboratory microcosms but contain the complexity and biologically realistic variation found in the natural aquatic ecosystems (Srivastava et al., 2004). In particular, food webs from bromeliad phytotelmata have been used to investigate global environmental changes, including the impacts of climate change, nutrient enrichment, drought, and other stressors on community structure, biodiversity, and ecosystem functioning (Antiqueirae, Petchey, Piccin, et al., 2018; Busse et al., 2018; Kratina et al., 2017; Petermann, Kratina, et al., 2015; Rezende et al., 2021; Romero et al., 2020; Srivastava et al., 2020; Teixeira et al., 2018). Tank bromeliads also provision a suite of important ecosystem functions and services, such as supporting local biodiversity, regulating the water dynamics and spread of disease, capturing greenhouse gasses, and cycling of nutrients (Ladino et al., 2019).

In this study, we experimentally warmed naturally assembled aquatic communities occurring within 50 tank bromeliads to quantify the impacts of warming on the structure of tropical freshwater aquatic communities. We tested how warming affects the density, local species richness, and beta diversity components of different groups of microflora and microfauna. We predict that warming would have stronger impact of microfauna communities than microflora communities because larger organisms are more sensitive to warming than smaller organisms (Brown et al., 2004). We hypothesized that: (i) warming would reduce local species richness (alpha diversity), but increase beta diversity, mainly through richness difference (*β*_rich_); (ii) warming would preferentially reduce the alpha diversity and density of some groups of the aquatic communities, especially larger organisms such as copepods.

## 2 MATERIALS AND METHODS

### 2.1 Study site and experimental design

**FIGURE 1:**
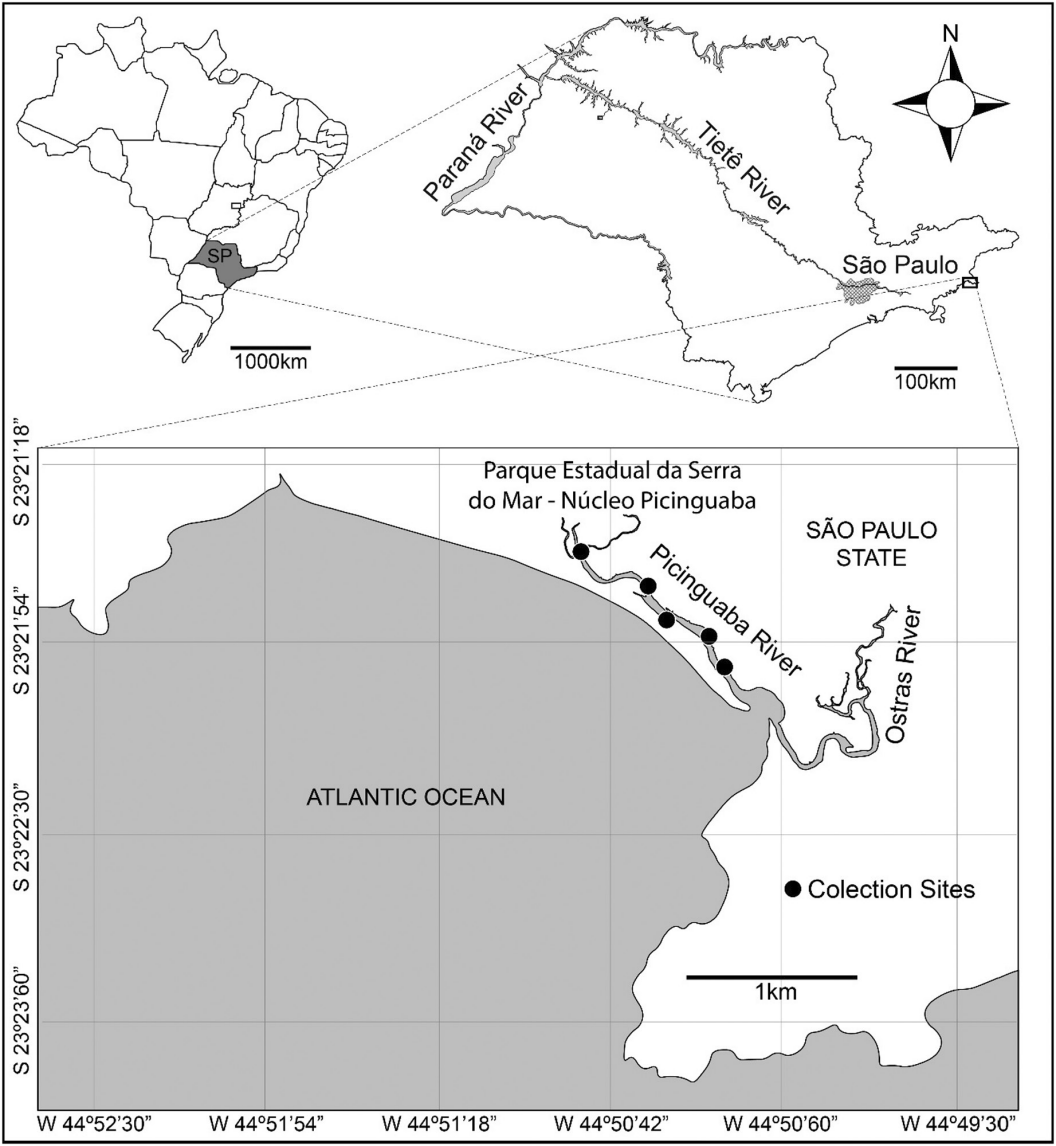
Study area showing the sampling sites of the bromeliads used in the experiment, located in the Parque Estadual Serra do Mar, Núcleo Picinguaba, São Paulo‐SP.

The study was carried out in the Parque Estadual Serra do Mar, Núcleo Picinguaba, one of the largest remaining fragments of Atlantic Forest in south‐eastern Brazil (approximately 47,500 ha), on the coast of São Paulo state (Figure 1). We collected samples for our field experiment from a Restinga forest, coastal Atlantic Forest vegetation characterized by herbs, shrubs and low trees, poor, sandy, and acidic soils (Araujo, 1992; Gomes et al., 2007), and high numbers of endemic species (Marques et al., 2015). The Atlantic rainforest is one of the most threatened biodiversity hotspots globally (Laurance, 2009), and Restingas are among the most vulnerable habitats within them (Marques et al., 2015), being particularly threatened by climate change (Inague et al., 2021).

Tank‐bromeliads are diverse and common in Restingas, where they act as biodiversity amplifiers (Cogliatti‐Carvalho et al., 2010; Ladino et al., 2019; Rocha et al., 2004). We selected the tank‐bromeliad *Neoregelia johannis* as the model system for our warming experiment as it is a large, dominant species in the region. Individual *N. johannis* hold almost 2 L of water on average, majorly contributing to freshwater habitat provision in an environment where standing water is rare (Antiqueira, Petchey, & Romero, 2018; Cogliatti‐Carvalho et al., 2010). These aquatic ecosystems held within tank‐bromeliads house diverse, multitrophic communities of macroinvertebrates and microorganisms (Petermann, Farjalla, et al., 2015; Petermann, Kratina, et al., 2015), with strong impacts on ecosystem function both within and outside of the bromeliads (Ladino et al., 2019; Leroy et al., 2016). This complexity along with their large phytotelmata which allows for easy access and manipulation of the aquatic communities, means *N. johannis* has been widely used as a model system for testing ecological hypotheses using naturally assembled communities under controlled conditions (Antiqueira, Petchey, Piccin, et al., 2018; Antiqueira, Petchey, & Romero, 2018; Migliorini & Romero, 2020; Nash et al., 2021).

We collected 50 individual *N. johannis* within a 1 km^2^ patch of Restinga forest. Bromeliads were collected at ~1.25 m (±0.5 m) from the forest floor, to eliminate height stratification (Kratina et al., 2017) and salinity effects. All 50 bromeliads were then left for 5–10 days at a single location within the collection area to acclimatize to the same conditions and for some natural colonization of microorganisms to occur, allowing for maximum natural homogenization of the communities before the experiment. Hereafter, the bromeliads were translocated to a nearby, outdoor, experimental plot, maintaining the naturally assembled communities as found in their natural environment (further methodological details in Nash et al., 2021).

The bromeliads were individually enclosed in protective netting which allowed for natural abiotic fluctuations in temperature and rainfall but avoided disruption from biotic factors such as falling organic material or animals. Bromeliad size is one of the most important determinants of phytotelmata community structure (Petermann, Farjalla, et al., 2015; Petermann, Kratina, et al., 2015). To account for this variation and ensure that our experimental temperature gradient was evenly spread across different bromeliad sizes after randomization, the plants were divided into five similar size categories (as in Antiqueira, Petchey, Piccin, et al., 2018; Nash et al., 2021). Thus, each experimental block consisted of 10 individual bromeliads from a similar size category randomly distributed along a temperature gradient. The experiment was conducted over 44 days to cover the full life cycles of most phytotelmata‐inhabiting invertebrates (Dézerald et al., 2017), and multiple generations of microorganisms, and capture the range of interactions between macro‐ and micro‐organisms (Bernabé et al., 2018). We carried out this study over March and April 2018, at the end of the rainy season, when phytotelmata‐inhabiting organism abundances are higher (Mestre et al., 2001) and environmental temperature and macrofaunal diversity are more stable (Busse et al., 2018).

To simulate future climate warming we used projections of temperature increase in Brazil for 2040 (+2°C) and 2100 (+4°C) (IPCC, 2022; PBMC, 2015) along with two higher levels (+6°C and +8°C) to simulate temperature extremes, as our four target warming levels. These were combined with the control treatment (ambient temperature, no warming) to create a temperature gradient of five target levels. To achieve the gradient, a custom system of sensors and aquarium heaters (1 W, 110 V) were inserted in two opposite lateral phytotelmata of each bromeliad, controlled by a central unit running the Total Control® software. The sensors recorded the bromeliad water temperature every 30 min, warming each bromeliad to its target level relative to the ambient (control) bromeliad in each block in real time. The ultimate continuous mean temperature gradient achieved spanned from 23.58 to 31.72°C, with a mean ambient temperature of 23.81°C ± 0.14 (mean ± SD). Precise measurements of bromeliad size (maximum water volume; L) and detrital contents (coarse particulate organic matter; g) were obtained at the end of the experiment to control and test for natural structural variation in the natural microcosms. Further methodological details on the bromeliad field experimental manipulation can be found in Nash et al. (2021).

### 2.2 Sample collection and laboratory analysis

We performed two sample collections of the aquatic communities, at the beginning and at the end of the experiment. We collected a homogeneous mixture of 50 mL of water from all 50 microecosystems. Subsequently, 1.5 mL of this sample was conditioned in a 2‐mL cryogenic tube. The rest was fixed with the Lugol's acetic fixative (5%) totaling, at the end of the sampling, 100 samples (50 samples from each collection period). The samples were subjected to quantitative analyses of specific groups (Antiqueira, Petchey, & Romero, 2018) of microflora (blue algae, green algae, diatoms, and myxotrophic flagellates) and microfauna (testaceous amoebae, ciliates, rotifers, and copepods). The microflora density and richness were estimated using an inverted microscope, using the Utermöhl method (Utermöhl, 1958), with a 40× magnification. Each 2 mL sample was diluted to 4 mL or more, according to the concentration of detritus. The sedimentation volume was 3 mL, and the sedimentation time was at least 3 h. A count of 50 fields was performed randomly. Density values were calculated according to APHA (1998), and species were identified according to specific literature (Bicudo & Menezes, 2017). Microfauna samples were stained with Rose of Bengal for direct analysis under an optical microscope using 200× magnification. The microfauna density was estimated by counting the individuals in Sedgewick‐Rafter chambers. Organisms were identified using specific taxonomic keys to the lowest possible taxonomic level (Foissner et al., 1999; Foissner & Berger, 1996; Koste, 1978; Reid, 1985; Souza, 2008).

### 2.3 Statistical analysis

We analyzed 50 community samples from the beginning and 50 community samples from the end of the experiment. The samples from the beginning of the experiment allowed us to establish the absence of baseline differences in aquatic communities at the start of the experiment across treatments. To assess how warming and volume affected the density and richness of microflora and microfauna (prediction i), and different taxonomic groups (prediction ii), multiple linear regressions were also fit to the data from the end of experiment. We included mean water temperature and volume as continuous predictor variables, final detrital mass as covariate, and their interaction, using the function “lm” from the stats package. We also performed the model comparison with the Akaike information criterion (AIC) to select the best model using the function “AIC” from the *stats* package. This analysis allowed us to test the impacts of warming on the aquatic communities and account for starting differences in their structural environment. When the assumption of homoscedasticity was not met, we transformed the response variable using log (for density) and square root (for richness).

To assess how warming altered total beta diversity and its two components of replacement (*β*_repl_) and difference in richness (*β*_rich_), the Jaccard distance matrix was analyzed with the method proposed by Podani and Schmera (2011), using the “beta” function of the BAT package (Cardoso et al., 2020). In order to determine any treatment‐related patterns in total beta diversity and its components, a distance‐based redundancy analysis (dbRDA; Legendre & Legendre, 2012) was applied using the function “dbrda” from the vegan package (Oksanen et al., 2019). This analysis allowed us to test the significance of the associations. All analyses were performed with the statistical analysis software R® (version 4.1.2; R Core Team, 2016).

## 3 RESULTS

At the end of the study, there were 59 species of aquatic communities, comprising 27 species of microflora and 32 species of microfauna. Microflora alfa diversity decreased by 11% from the initial number of species, whereas microfauna alfa diversity increased by 54% from the initial number of species. For the microflora, diatoms were the most dominant group with 10 species, followed by flagellates with 7 species, green algae with 6 species, and blue algae with only 4 species (Table S1). For the microfauna, ciliates were the most dominant group with 15 species, the most prevalent orders being Colpodea and Hymenostomatida, represented by 5 species each (Table S1). The testate amoebae were represented by 14 species, of which Arcellidae was the most representative order, with 4 species (Table S1). Three rotifer species and one copepod species were also recorded (Table S1).

**FIGURE 2:**
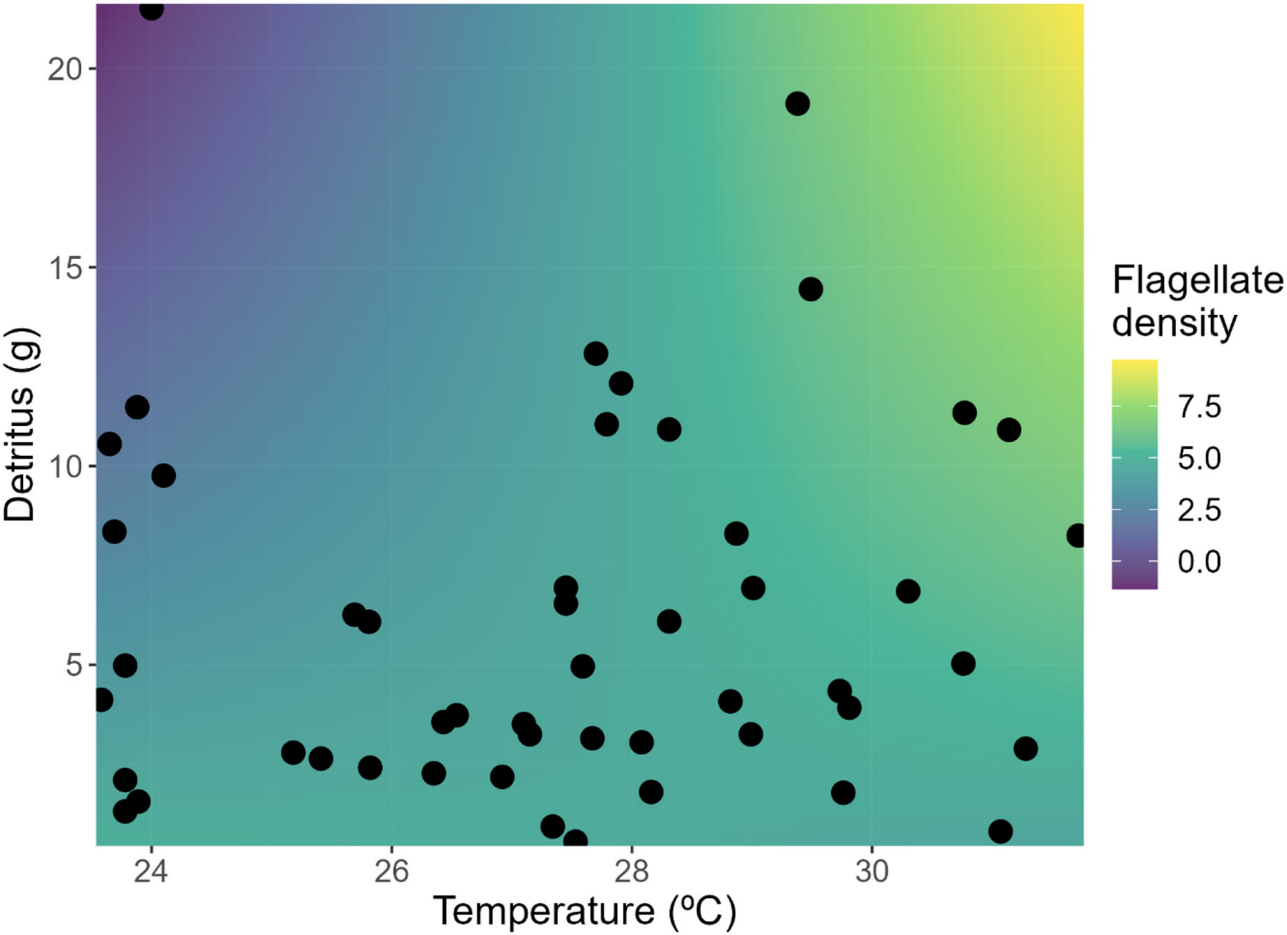
Surface plot illustrating the significant interaction between experimental temperature and detritus biomass on the density of flagellates. The positive impact of warming was strongest (the highest flagellate density indicated by yellow color) in bromeliads with high detritus biomass and continuously weakened (purple) as the temperature decreased. The color was assigned to the flagellate density response, predicted by our model using the visreg function (Breheny & Burchett, 2019).

**FIGURE 3:**
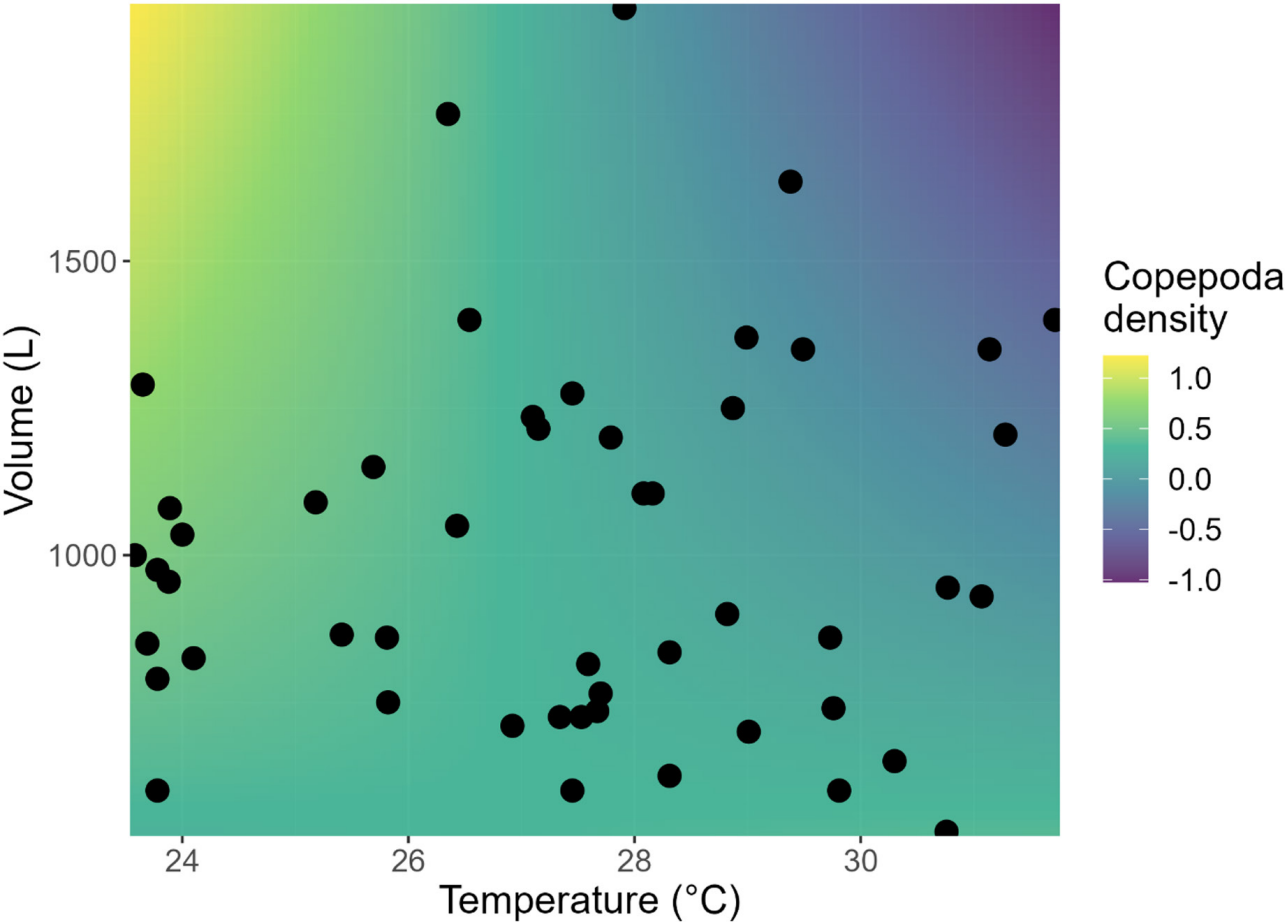
Surface plot illustrating the significant interaction between experimental temperature and bromeliad water volume on copepod density. The positive impact of water volume was strongest (the highest copepod density indicated by yellow color) in bromeliads with lowest temperature and continuously weakened (purple) as the volume and temperature increases. The color was assigned to the copepod density response, predicted by our model using the visreg function (Breheny & Burchett, 2019).

At the end of the experiment, warming, bromeliad water volume, and their interaction did not alter the total microflora density (Table 1) or total microflora richness (Table 2). However, there was a significant interactive effect of warming and detritus biomass covariate on flagellate density (*F*_1,42_ = 4.64, *p* = .020). More specifically, the highest density of flagellates was found in bromeliad ecosystems with the highest temperature and detritus amount (Figure 2). Furthermore, there was a significant negative effect of bromeliad volume on flagellate alpha diversity (*F*_1,46_ = −3.04, *p* = .005). Warming and bromeliad water volume did not alter the total density (Table 1) or total richness (Table 2) of aquatic microfauna. However, there was a significant interactive effect of warming and bromeliad water volume on copepod density (*F*_1,42_ = −4.97, *p* = .028). Specifically, the highest copepod density was found in bromeliads with higher water volume and low temperatures (Figure 3).

**TABLE 1:** The effects of warming, detritus, and bromeliad water volume on the density of microflora and microfauna groups at the end of the experiment.

Effect	*df*	Estimates	*F*	*p*
Microflora				
Total microflora				
Warming	1	0.070	0.176	.567
Volume	1	−0.001	1.637	.207
Detritus biomass	1	−0.120	5.508	.069
Residual	46			
Blue algae				
Warming	1	0.546	0.003	.830
Volume	1	−0.035	1.853	.083
Detritus biomass	1	−1.043	3.129	.445
Residual	46			
Green algae				
Warming	1	−0.043	0.059	.762
Volume	1	0.001	2.496	.121
Detritus biomass	1	−0.116	1.172	.139
Residual	46			
Diatoms				
Warming	1	−2.397	2.110	.145
Volume	1	0.006	0.258	.614
Detritus biomass	1	−0.101	0.001	.907
Residual	46			
Flagellates				
Warming × Detritus biomass	1	4.637	0.474	**.020**
Warming	1	0.212	1.398	.198
Volume	1	−0.001	1.869	.178
Detritus biomass	1	−0.053	1.158	.540
Residual	42			
Microfauna				
Total microfauna				
Warming	1	−2.663	0.385	.584
Volume	1	5.983	0.000	.987
Detritus biomass	1	−5.452	4.886	**.040**
Residual	46			
Testate amoebae				
Warming	1	−3.292	0.660	.405
Volume	1	5.329	0.028	.866
Detritus biomass	1	9.188	0.262	.663
Residual	46			
Ciliates				
Warming	1	−0.013	0.103	.744
Volume	1	0.000	0.633	.430
Detritus biomass	1	−0.029	1.347	.185
Residual	46			
Rotifera				
Warming	1	0.039	0.473	.426
Volume	1	−0.060	0.142	.707
Detritus biomass	1	−0.000	6.599	**.023**
Residual	46			
Copepoda				
Warming × Volume	1	−4.971	1.409	**.028**
Warming	1	−5.743	4.318	**.045**
Volume	1	−8.825	0.155	.695
Detritus biomass	1	4.293	0.031	.775
Residual	42			

*Note*: The summary statistics from the linear regressions. Values in bold represent significant relationships with *p* < .05.

**TABLE 2:** The effects of warming, detritus, and bromeliad water volume on the species richness (alpha diversity) of microflora and microfauna groups at the end of the experiment.

Effect	df	Estimates	*F*	*p*
Microflora				
Total microflora				
Warming	1	−0.047	0.360	.698
Volume	1	−0.001	3.399	.071
Detritus biomass	1	−0.143	8285	**.033**
Residual	46			
Blue algae				
Warming	1	−0.006	0.125	.858
Volume	1	−0.0004	3.273	.077
Detritus biomass	1	−0.018	2.638	.319
Residual	46			
Green algae				
Warming	1	−6.368	1.395	.240
Volume		7.435	0.030	.863
Detritus biomass	1	−1.096	0.000	.969
Residual	46			
Diatoms				
Warming	1	−0.021	0.148	.694
Volume	1	0.0002	0.389	.535
Detritus biomass	1	−0.026	0.553	.374
Residual	46			
Flagellates				
Warming	1	0.099	1.491	.149
Volume	1	−0.028	3.042	**.005**
Detritus biomass	1	−0.001	8.623	.436
Residual	46			
Microfauna				
Total microfauna				
Warming	1	−0.050	0.319	.572
Volume	1	0.000	0.724	.399
Detritus biomass	1	−0.086	2.652	.077
Residual	46			
Testate amoebae				
Warming	1	0.011	0.056	.834
Volume	1	0.000	0.141	.708
Detritus biomass	1	−0.001	0.006	.972
Residual	46			
Ciliates				
Warming	1	0.011	0.108	.744
Volume	1	0.000	0.323	.572
Detritus biomass	1	−0.021	0.989	.269
Residual	46			
Rotifera				
Warming	1	0.027	0.370	.516
Volume	1	0.000	0.109	.742
Detritus biomass	1	−0.041	3.431	.068
Residual	46			

*Note*: Values in bold represent significant relationships with *p* < .05.

**FIGURE 4:**
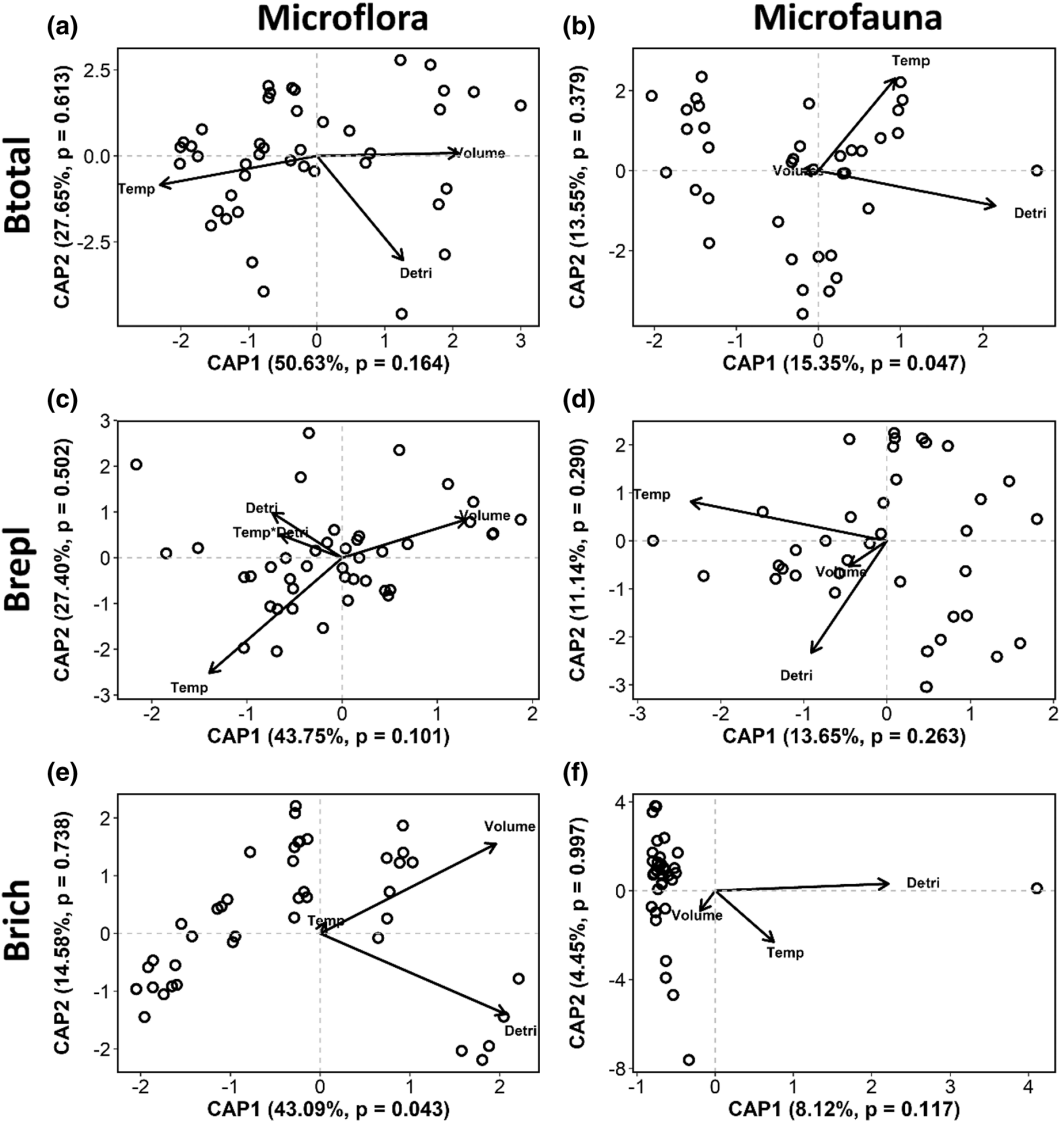
Distance‐based redundancy analysis for *β*_total_ (a) microflora and (b) microfauna; *β*_repl_ (c) microflora and (d) microfauna; *β*_rich_ (e) microflora and (f) microfauna illustrating the dissimilarity response. The dbRDA1 and dbRDA2 represent the first and second constraint axes generated by the analysis, respectively. Detri is detritus mass; Temp is mean temperature; Volume is total bromeliad water volume; Temp * Detri is the interactive effect between mean temperature and detritus mass.

The *β*_repl_ component of microflora communities was significantly influenced by the interaction between experimental warming and detritus biomass (*β*_repl_; Table 3; Figure 4). *β*_repl_ differed in warmer bromeliads with high detritus biomass in comparison to warm bromeliads with low detritus biomass (Figure 4). The *β*_rich_ diversity component of the microflora and microfauna were significantly affected by detritus biomass (Table 3; Figure 4). However, the microfauna *β*_repl_ diversity was negatively influenced by experimental warming (Table 3; Figure 4). In relation to the other components of the beta diversity, there was no effect of treatments on both microflora and microfauna.

**TABLE 3:** The effects of warming, bromeliad volume, and detritus biomass on dissimilarity in algal and microfauna composition in tank bromeliads.

Effect	df	Estimates	*F*	*p*
Microflora				
*β*_total_				
Warming	1	−0.393	1.556	.095
Volume	1	0.310	1.386	.139
Detritus biomass	1	−0.151	1.351	.152
*β*_repl_				
Warming x Detritus biomass	1	−2.202	1.804	**.035**
Warming	1	−0.291	1.468	.095
Volume	1	0.318	1.252	.211
Detritus biomass	1	−0.224	0.889	.542
*β*_rich_				
Warming	1	0.012	0.167	.996
Volume	1	0.668	2.081	.087
Detritus biomass	1	0.733	3.492	**.015**
Microfauna				
*β*_total_				
Warming	1	0.380	1.559	.086
Volume	1	−0.171	0.971	.439
Detritus biomass	1	0.633	2.099	**.016**
*β*_repl_				
Warming	1	−0.743	1.645	**.050**
Volume	1	−0.129	0.802	.695
Detritus biomass	1	0.187	1.454	.089
*β*_rich_				
Warming	1	0.222	0.588	.592
Volume	1	−0.063	0.765	.461
Detritus biomass	1	0.643	3.286	**.026**

*Note*: Values in bold represent significant relationships with *p* < .05. *β*_total_, total beta diversity; *β*_repl_, beta replacement diversity; *β*_rich_, beta diversity richness difference.

## 4 DISCUSSION

Our results showed that experimental warming and bromeliad water volume did not affect the total density and richness of microflora and microfauna, in contrast to our first hypothesis. However, both these factors negatively affected the total density of copepods and flagellate density. This result partly corroborated our second hypothesis, that warming would reduce the alpha diversity and density of some groups of the aquatic communities, especially larger organisms such as copepods. Warming aquatic ecosystems by 3°C has been shown to change planktonic food webs to be more dominated by fast‐growing species with small body sizes and rapid reproduction rates (Rasconi et al., 2015). These changes can alter biological interactions and cascade to other food web compartments (Vidussi et al., 2011). In addition, bromeliad water volume, which is a proxy for habitat size of bromeliad biota (Antiqueira, Petchey, & Romero, 2018; Petermann, Farjalla, et al., 2015), also negatively affected flagellate alpha diversity.

For aquatic organisms in general, the geographic range distribution is negatively associated with body size (e.g., Bie et al., 2012; Lansac‐Tôha et al., 2021; Padial et al., 2014). The negative effect of warming on the larger organisms (e.g., copepods) agrees with previous work showing that large‐bodied organisms may be disproportionately more negatively affected by rising environmental temperatures (Daufresne et al., 2009; Evans et al., 2019; Sheridan & Bickford, 2011). Thus, global warming, predicted over future decades, may reduce copepod densities by shifting environmental conditions beyond their physiological tolerance limits (Almén et al., 2014). As copepods are among the major components of lake and marine plankton communities, understanding the responses of this group to warming provides important insights into the functioning of freshwater ecosystem in general (Evans et al., 2019; Perbiche‐Neves et al., 2019). Moreover, copepods inhabiting tropical freshwaters may be strongly impacted by warming due to their high rates of endemism (2558 of 2814 species, or 90.9%), especially in the Neotropics with over 80% endemism (Boxshall & Defaye, 2008). Spatial and climatic variables may explain the high endemism rates of copepods because changes in environmental temperature regimes act as climatic filters, and they are likely to experience local and regional extinctions in the future warmer environments (Perbiche‐Neves et al., 2019).

Copepods comprise the dominant zooplankton taxa in most water bodies worldwide, and are one of the most important bioindicators for globally (Magouz et al., 2021). Ecologically, copepods are an important prey in the diets of commercially important larval, juvenile fish, and invertebrates, playing key roles in the transfer of organic matter (Atul & Rumana, 2021; Marcus, 2004; Montero et al., 2021). Because of this function, copepods can be used as a natural food for fish and as a viable alternative of high quality and easily digestible food (Atul & Rumana, 2021). However, the copepod density in bromeliads was strongly influenced by the combined effects of warming and bromeliad water volume. Similarly, Dézerald et al. (2014) found that bromeliad water volume (a proxy for habitat size) was strongly correlated with invertebrate richness and abundance, within larger habitats hosting more species, and richer species in the habitats contained higher proportions of large‐bodied predators. This indicates that greater predation pressure on copepods could be positively related to habitat size and intensify the negative effect of warming.

We did not observe any individual effect of warming on algae density or diversity. The optimal growth temperature for most microalgae ranges between 22 and 35°C (Singh & Singh, 2015), depending on the latitude and taxon‐specific temperature sensitivities (Thomas et al., 2016). Therefore, even at the worst temperature scenario, algae density may be not properly limited. Temperatures that exceed the thermal tolerance limits negatively affect microflora through degradation and lysis of their cells (Akimov & Solomonova, 2019). It has been previously found that bromeliad amoeba, algae, ciliates, and microfauna predators all had a unimodal relationship with environmental temperature (Kratina et al., 2017). However, there may be weaker effects on algae if the warming does not exceed 3, or 5–8°С for green algae (Akimov & Solomonova, 2019). Because bromeliads can experience high diurnal fluctuations in environmental conditions, their microalgae may have wider thermal tolerance limits (Pett‐Ridge & Silver, 2002), possibly explaining the weak impact of warming on algal density and richness observed here. Although tropical plankton are thought to be well adapted to high temperatures, it is critical to investigate responses of tropical phytoplankton communities to combined effects of warming and nutrient enrichment (Halac et al., 2013).

Community composition responses to warming differed among ecological groups of the aquatic communities. However, contrary to our hypothesis, the replacement of microfauna species (*β*_repl_) was deriving the changes in beta diversity with the experimental warming, that is, bromeliads with higher mean temperatures had more distinct composition, probably due to the occurrence of species more adapted to the warmer environment. Species replacement tends to be more common in the Neotropics, relative to temperate regions, due to relatively constant temperatures, limited acclimation potential, and narrower climate tolerances (Bennett et al., 2021; Buckley & Jetz, 2008; Sheldon et al., 2018). The close association between environmental temperature and species replacement in tropical regions suggests that species in these regions may be particularly susceptible to climate change (Buckley & Jetz, 2008). An important implication is that ecosystems with high rates of replacement require greater conservation focus to encompass the full range of community composition variation (Perbiche‐Neves et al., 2019). This result suggests that climate change can exclude more sensitive species while favoring those that are better adapted (Busse et al., 2018; Kratina et al., 2017). However, this selection of more adapted species can generate long‐term negative community consequences (Tundisi & Tundisi, 2008), such as the loss of functional redundancy and essential functional groups such as predators.

The interaction between warming and detritus biomass governed the microflora species substitution (*β*_repl_). This is because bacteria can support algal growth by recycling nutrients while also competing for essential nutrients and this can mediate remineralization and carbon sequestration (Buchan et al., 2014; Zou et al., 2016). However, the beta diversity of microflora was positively associated with detritus biomass, mainly due to the difference in the richness component (i.e., related to species loss). Considering that the diversity of insect predators of microfauna is positively related to detritus (Torreias & Ferreira‐Keppler, 2011) and these insects are negatively related with microfauna (Amadeo et al., 2017; Antiqueira, Petchey, & Romero, 2018), detritus could lead to increased predation pressure on prey aquatic communities, such as algae and rotifers. At the local scale, these species losses would have negative implications for ecosystem functioning and stability (Isbell et al., 2017; Rezende et al., 2021).

This study provides new experimental evidence that warming can alter the diversity of aquatic communities in the tropics. However, to draw more general conclusions it would be fruitful to explore a long‐term effect of experimental manipulation, focusing on the process of species succession. The future study of both resistance and resilience of aquatic communities to warming would help to characterize more sensitive organismal groups and, consequently, possible functional losses. Future research should investigate these responses of aquatic communities across a wider range of experimental temperatures, including the heatwave periods (Vad et al., 2022). The microbial communities are still poorly studied groups, and our results showed that the temperature promoted the change in the identity of the microfauna species. We also demonstrate that experimental warming enhanced beta diversity, mainly by replacing species (*β*_repl_) from upper trophic level groups (e.g., copepods). Such changes in trophic structure can alter important ecosystem processes such as decomposition and primary productivity. By limiting energy transfer to higher trophic levels, through negatively affecting microflora and microfauna, warming has both direct and indirect effects on the entire food webs. We argue that due to the controlled and replicated nature of this field experiment, our findings may be applicable to a wide range of aquatic environments in the tropics, most of which are experiencing changes in temperature regimes. Our findings suggest that large taxa that are strongly affected by climate warming should by prioritized for targeted conservation actions.

## AUTHOR CONTRIBUTIONS

**Melissa Progênio:** Conceptualization (equal); data curation (equal); formal analysis (equal); methodology (equal); visualization (equal); writing – original draft (equal); writing – review and editing (equal). **Pablo A. P. Antiqueira:** Conceptualization (equal); data curation (equal); writing – original draft (equal); writing – review and editing (equal). **Felipe R. Oliveira:** Conceptualization (equal); methodology (equal); writing – original draft (equal); writing – review and editing (equal). **Bianca R. Meira:** Conceptualization (equal); writing – original draft (equal); writing – review and editing (equal). **Fernando M. Lansac‐Toha:** Data curation (equal); formal analysis (equal); writing – review and editing (equal). **Luzia C. Rodrigues:** Methodology (equal); visualization (equal); writing – review and editing (equal). **Gustavo Q. Romero:** Conceptualization (equal); methodology (equal); supervision (equal); writing – review and editing (equal). **Liam N. Nash:** Conceptualization (equal); data curation (equal); formal analysis (equal); methodology (equal); writing – review and editing (equal). **Pavel Kratina:** Conceptualization (equal); data curation (equal); methodology (equal); supervision (equal); visualization (equal); writing – review and editing (equal). **Luiz F. M. Machado‐Velho:** Conceptualization (equal); methodology (equal); supervision (equal); writing – original draft (equal); writing – review and editing (equal).

## CONFLICT OF INTEREST STATEMENT

The authors declare that they have no conflict of interest.

## Supporting information


Table S1

## Data Availability

The data used in this study will be archived in the public archive Dryad (http://datadryad.org).
