# Supplementary material for: Effects of warming on the structure of aquatic communities in tropical bromeliad microecosystems
Source: Ecol Evol. 2023 Feb 22;13(2):e9824. doi: 10.1002/ece3.9824 (PMC9944163; doi:10.1002/ece3.9824)
Supplement: Supplementary file 1 — Table S1 [file ECE3-13-e9824-s001.docx]

**Supplementary material**

**Table S1.** A list of microflora and microfauna species found in the experiment. (NI)= Not Identified.

| **Groups of the microbiota** | **Taxonomic groups** | **Taxonomic identification** |
| --- | --- | --- |
| **Blue algae** | Cyanophyceae | Cianobactéria 1 (NI) |
|  |  | Cianobactéria 2 (NI) |
|  |  | Pseudanabaenaceae (NI) |
|  |  | *Romeria* sp. Koczwara, 1932 |
|  |  |  |
| **Green algae** | Chlorophyceae | Chlorophyta (NI) |
|  |  |  |
|  | Oedogoniales | *Oedogonium reinschii* J.Roy ex Hirn |
|  |  | *Oedogonium* sp1 |
|  |  | *Oedogonium* sp2 |
|  |  | *Rhopalosolen* sp. |
|  |  |  |
|  | Trebouxiophyceae | *Crucigenia* sp. |
|  |  |  |
| **Diatoms** | Bacillariophyceae | *Achnanthidium* sp. |
|  |  | *Centrales* 1 (NI) |
|  |  | *Cocconeis* sp. |
|  |  | *Eunotia* sp. |
|  |  | *Luticola* sp. |
|  |  | *Navicula* sp. |
|  |  | *Nitzschia* sp. |
|  |  | Pennales 1 (NI) |
|  |  | Pennales 2 (NI) |
|  |  | *Pinnularia* sp. |
|  |  |  |
| **Flagellates** | Cryptophyceae | *Cryptomonas* sp1 |
|  |  | *Cryptomonas* sp2 |
|  |  |  |
|  | Euglenophyceae | *Euglena* encistada |
|  |  | *Euglena* sp1 |
|  |  | *Euglena* sp2 |
|  |  | *Euglena* sp3 |
|  |  | *Euglena* sp4 |
|  |  |  |
| **Testate amoebae** | Arcellidae | *Arcella discoides* Ehrenberg*,*1871 |
|  |  | *Arcella megastoma* Pénard, 1913 |
|  |  | *Arcella mitrata* Leidy, 1879 |
|  |  | *Arcella vulgaris* Ehrenberg,1830 |
|  |  |  |
|  | Centropyxidae | *Centropyxis aculeata* Ehrenberg,1830 |
|  |  | *Centropyxis ecornis* Ehrenberg, 1841 |
|  |  | *Centropyxis minuta* Deflandre, 1929 |
|  |  |  |
|  | Difflugidae | *Difflugia lobostoma* Leidy, 1877 |
|  |  | *Difflugia* cf. *minuta* Rampi, 1950 |
|  |  | *Netzelia wailesi* (Ogden, 1980) Meisterfeld, 1984 |
|  |  |  |
|  | Euglyphidae | *Euglypha strigosa* Ehrenberg,1871 |
|  |  |  |
|  | Nebelidae | *Pseudonebela* sp. |
|  |  |  |
|  | Phryganellidae | *Phryganella hemisphaerica* Pénard, 1902 |
|  |  |  |
| **Ciliates** | Colpodea | *Colpoda cucullus* (Müller, 1773) Gmelin, 1790 |
|  |  | *Colpoda ecaudata* (Liebmann, 1936) Foissner et al., 1991 |
|  |  | *Colpoda inflata* (Stokes, 1884) Kahl, 1931 |
|  |  | *Colpoda steinii* Maupas, 1883 |
|  |  | *Platyophrya vorax* Kahl, 1926 |
|  |  |  |
|  | Heterotrichida | *Metopus* spp. sensu lato |
|  |  |  |
|  | Hymenostomatida | *Dexiostoma campylum* (Stokes, 1886) Jankowski, 1967 |
|  |  | *Glaucoma* sp. |
|  |  | Hymenomastida 1 (NI) |
|  |  | *Uronema nigricans* (Müller, 1786) Florentin, 1901 |
|  |  | *Sathrophilus muscorum* (Kahl, 1931) Corliss, 1960 |
|  |  |  |
|  | Oligotrichida | *Halteria grandinella* (Müller, 1773) Dujardin, 1841 |
|  |  |  |
|  | Prostomatida | *Coleps hirtus* (Müller, 1786) Nitzsch, 1827 |
|  |  | *Coleps spetai* Foissner, 1984 |
|  |  | Prostomatida 1 (NI) |
|  |  |  |
| **Rotifers** | Lecanidae | *Lecane* sp1 |
|  |  | *Lecane* sp2 |
|  |  |  |
|  | Lepadellidae | *Lepadella* sp. |
|  |  |  |
| **Copepods** | Cyclopoida | Cyclopoida 1 (NI) |
